# Non-covalent SARS-CoV-2 M^pro^ inhibitors developed from in silico screen hits

**DOI:** 10.1038/s41598-022-06306-4

**Published:** 2022-02-15

**Authors:** Giacomo G. Rossetti, Marianna A. Ossorio, Stephan Rempel, Annika Kratzel, Vasilis S. Dionellis, Samia Barriot, Laurence Tropia, Christoph Gorgulla, Haribabu Arthanari, Volker Thiel, Peter Mohr, Remo Gamboni, Thanos D. Halazonetis

**Affiliations:** 1 Department of Molecular Biology, University of Geneva, 1205 Geneva, Switzerland; 2 FoRx Therapeutics AG, 4056 Basel, Switzerland; 3 Institute of Virology and Immunology, University of Bern, 3012 Bern, Switzerland; 4 Department of Infectious Diseases and Pathobiology, Vetsuisse Faculty, University of Bern, 3012 Bern, Switzerland; 5 Graduate School for Cellular and Biomedical Sciences, University of Bern, Bern, Switzerland; 6 Department of Biological Chemistry and Molecular Pharmacology, Harvard Medical School, Harvard University, Boston, MA 02115 USA; 7 Department of Physics, Faculty of Arts and Sciences, Harvard University, Cambridge, MA 02138 USA; 8 Department of Cancer Biology, Dana-Farber Cancer Institute, Boston, MA 02115 USA; 9 NANDASI Pharma Advisors, 4123 Allschwil, Switzerland

**Keywords:** X-ray crystallography, Screening, Small molecules

## Abstract

M^pro^, the main protease of the severe acute respiratory syndrome coronavirus 2 (SARS-CoV-2), is essential for the viral life cycle. Accordingly, several groups have performed in silico screens to identify M^pro^ inhibitors that might be used to treat SARS-CoV-2 infections. We selected more than five hundred compounds from the top-ranking hits of two very large in silico screens for on-demand synthesis. We then examined whether these compounds could bind to M^pro^ and inhibit its protease activity. Two interesting chemotypes were identified, which were further evaluated by characterizing an additional five hundred synthesis on-demand analogues. The compounds of the first chemotype denatured M^pro^ and were considered not useful for further development. The compounds of the second chemotype bound to and enhanced the melting temperature of M^pro^. The most active compound from this chemotype inhibited M^pro^ in vitro with an IC_50_ value of 1 μM and suppressed replication of the SARS-CoV-2 virus in tissue culture cells. Its mode of binding to M^pro^ was determined by X-ray crystallography, revealing that it is a non-covalent inhibitor. We propose that the inhibitors described here could form the basis for medicinal chemistry efforts that could lead to the development of clinically relevant inhibitors.

## Introduction

In December 2019, the Chinese health authorities reported the emergence of a disease, now referred to as COVID-19, that was declared a pandemic by the World Health Organization just a few months later^1^. The causative agent of COVID-19 is a novel coronavirus named SARS-CoV-2^2^.

Similar to other coronaviruses, SARS-CoV-2 is an enveloped, positive-sense, single-stranded RNA virus. Its genome contains at least nine open reading frames (ORFs), of which ORF1a and ORF1b give rise to polyproteins pp1a and pp1ab, respectively^3^. A main protease (M^pro^, also called 3C-like protease) and a papain-like protease (PL^pro^) cleave these polyproteins into characteristic nonstructural proteins^4^. M^pro^ is essential for the viral life cycle, as it is needed to generate the mature forms of most of the nonstructural proteins of the virus; it contains a cysteine-histidine dyad at its catalytic center and cleaves its substrates at sites comprising a glutamine followed by a residue with a small side chain. M^pro^ is highly conserved within the coronavirus family and is considered a good target for the development of drugs that could treat COVID-19 patients^5–7^.

Most efforts to identify M^pro^ inhibitors have focused on repurposing or modifying inhibitors of M^pro^-related proteases^8–19^. Among the most potent of these inhibitors are GC376, boceprevir/telaprevir analogues and the PF-00835231 and PF-07321332 compounds; they are all irreversible inhibitors that form a covalent bond with the thiol group of the catalytically important residue Cys145. GC376, originally developed as a Norwalk virus 3CL^pro^ inhibitor^20,21^, inhibits SARS-CoV-2 M^pro^ in vitro with a half maximal inhibitory concentration (IC_50_) of 30 ± 8 nM^12,13^. Analogues of boceprevir and telaprevir, two inhibitors approved for the treatment of hepatitis C, inhibit M^pro^ with IC50 values as low as 7.6 ± 0.1 nM in vitro and show activity in a mouse model of SARS-CoV-2 infection^22^. Finally, PF-00835231 and PF-07321332 are related compounds derived from an inhibitor of the M^pro^ of SARS-CoV-1^14,15^. PF-00835231 requires continuous intravenous infusion as a prodrug to achieve effective doses in the plasma of human patients^23^, while PF-07321332 can be administered orally and promises to have a significant impact on the course of the COVID-19 pandemic^15^.

De novo M^pro^ inhibitors have been identified by either in silico or physical screens. Three in silico screening studies are particularly relevant here: two studies that ranked more than one billion compounds each, but did not validate the identified hits^24,25^, and a third study that ranked 6.5 million compounds and validated seven top compounds, of which the most potent exhibited an IC50 of 4.2 μM in vitro^26^. Among the studies that employed physical screens to identify M^pro^ inhibitors, one study screened a small library of chemical fragments for binding to M^pro^ by X-ray crystallography and identified several hits, which, however, were not developed further^27^. A second study screened a DNA-encoded library and identified compounds that inhibited M^pro^ with inhibition constants in the 35–45 nM range^28^. These inhibitors bound to M^pro^ covalently, as seen with the repurposed inhibitors, presumably because reactive, electrophilic compounds were included in the DNA-encoded library.

Covalent inhibitors often require extensive development to limit off-target effects and associated toxicity. Therefore, the development of non-covalent M^pro^ inhibitors, as an alternate strategy, is also worth pursuing. Here we characterized selected hits from the two very large in silico screens cited above^24,25^. The vast majority of the examined hits did not bind to or inhibit M^pro^ in vitro. However, a handful of hits were validated and these served as starting points for the development of a non-covalent M^pro^ inhibitor that was capable of inhibiting SARS-CoV-2 replication in tissue culture cells.

## Results

### Validation of putative SARS-CoV-2 M^pro^ inhibitors identified by in silico screens

To validate candidate inhibitors of SARS-CoV-2 M^pro^ identified by in silico screens, we utilized an in vitro protease activity assay. An expression plasmid encoding the M^pro^ protease with a C-terminal His-tag^8^ was introduced in the *E. coli* strain BL21-Gold (DE3) and recombinant M^pro^ was expressed and purified by affinity and size exclusion chromatography (Supplementary Fig. S1A). Protease activity was determined using a fluorescence resonance energy transfer (FRET) assay. The substrate peptide contained at its N-terminus a fluorescent dye (HiLyte-Fluor488) and at its C-terminus a quencher (QXL520); cleavage of the peptide by M^pro^ led to an increase in fluorescence intensity. The protease and fluorogenic substrate were assayed at 22 °C at final concentrations of 100 nM and 500 nM, respectively. Fluorescence intensity was measured every 10 min; in the absence of an M^pro^ inhibitor, fluorescence intensity increased linearly during the first 60 min of the reaction, whereas in the presence of the potent GC376 inhibitor^12,13^ no increase in fluorescence intensity was observed (Supplementary Fig. S1B).

We examined hits from two in silico screening studies^24,25^ that ranked the docking of molecules present in the REAL Space or ZINC chemical compound libraries^29,30^. In the first study, two in silico screens were performed, using two different three-dimensional structures to encompass the conformational flexibility of the active site of M^pro^^24^. The first screen, referred to as screen 1A, used the structure of M^pro^ described by Jin et al.^10^ (pdb id: 6lu7) as target, while the second screen, referred to as screen 1B, used the structure of Dai et al.^9^ (pdb id: 6m0k) with minor changes in the side-chain rotamers of residues Ser46, Met49 and Cys145 to capture a more open conformation of the active site. The two screens docked the same library of compounds; nevertheless, comparison of the top 1,000 hits of each screen, revealed an overlap of only 12 compounds. The second study performed one in silico screen, hereafter referred to as screen 2, of 1.3 billion compounds^25^, using the structure of M^pro^ described by Jin et al.^10^ (pdb id: 6lu7) as target.

From the 3,808 top-ranking compounds of screen 1A, 195 compounds were manually selected for on-demand synthesis aiming for chemical diversity and drug-like features (Supplementary Table 1); whereas, from the 3,851 top-ranking compounds of screen 1B, 226 compounds were selected for on-demand synthesis (Supplementary Table 2). In addition, guided by the results of a crystallographic fragment screen^27^ that showed a fragment containing a nitrile group deep in the active site of M^pro^ (pdb id: 5r82), we identified all the nitrile-containing compounds among the top 20,000 hits of screens 1A and 1B. This list included 253 compounds, 45 of which were selected for on-demand synthesis (Supplementary Table 3). Twelve of the 15 top-ranking compounds from screen 1A were also selected for on-demand synthesis (Supplementary Table 4). Finally, from screen 2, eight of the 15 top-ranking hits were selected for on-demand synthesis (Supplementary Table 4).

**Figure 1:**
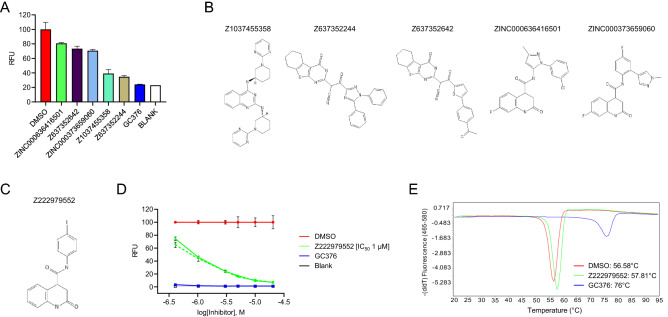
Development of M^pro^ inhibitors from in silico screening hits. (**A**) Graph showing the inhibitory activity of the five validated in silico screening hits tested at a final concentration of 40 μM in an in vitro protease-activity assay. After addition of the FRET substrate, fluorescence was acquired at 10 min intervals over 60 min. For each compound, the increase in fluorescence intensity was normalized to the DMSO control. GC376, a previously described M^pro^ inhibitor, served as a positive control. Blank, reaction omitting M^pro^; RFU, relative fluorescence units. (**B**) Chemical structure of the five validated compounds. (**C**) Chemical structure of Z222979552, the most active dihydro-quinolinone compound obtained after two rounds of chemical structure similarity searches of the REAL space library of molecules. (**D**) Dose–response curves for compound Z222979552 examined at 0.4, 1, 3, 5, 10 and 20 μM final compound concentrations, in absence (solid lines) or presence (dashed lines) of 0.1 μg protein lysate. DMSO, GC376 and blank controls are as described above. (**E**) Thermal shift assay performed in the presence of DMSO or 20 μM of compounds Z222979552 or GC376. The graphs show the derivatives of the melting curves used to calculate the melting temperature of M^pro^.

In total, we had 486 compounds synthesized and all these compounds were assayed at a final concentration of 40 μM for their ability to inhibit the protease activity of M^pro^. Remarkably, only five compounds inhibited M^pro^ more than the pre-defined threshold level of 25% inhibition (Fig. 1A). The active compounds were: the diamino-quinazoline Z1037455358 (Fig. 1B), which is one of the 226 compounds selected from the 3,851 top-ranking compounds of screen 1B (Supplementary Table 2); the structurally-related nitriles Z637352244 and Z637352642 (Fig. 1B), which are two of the 45 nitrile-containing compounds selected from screens 1A and 1B (Supplementary Table 3); and the structurally-related dihydro-quinolinones ZINC000636416501 and ZINC000373659060 (Fig. 1B), which are two of the eight selected top-ranking compounds from screen 2 (Supplementary Table 4). All these five compounds were characterized further, as described below.

### Characterization of the diamino-quinazoline and nitrile compounds

We first focused our efforts on compound Z1037455358, which contains a diamino-quinazoline core (Fig. 1B). The IC_50_ of this compound in the protease assay was 26 μM (Supplementary Fig. S2A). Analogues of Z1037455358 were identified using similarity and substructure searches of the REAL Space library of chemical compounds^29^; 108 of these analogues were selected for on-demand synthesis (Supplementary Table 5); however, none of the analogues were more potent than the parent compound in the biochemical assay. Therefore, we retained only the parent compound for further analysis.

Next, we examined the two structurally-related compounds Z637352244 and Z637352642, both of which contain a nitrile group (Fig. 1B). A nitrile group is also present in a fragment that was found to bind M^pro^ by crystallographic screening^27^. To identify more potent compounds, we selected 301 analogues for on-demand synthesis (Supplementary Table 6) and examined them at a final concentration of 40 μM for their ability to inhibit M^pro^ in the protease assay. Five analogues were more active than the original compounds (Supplementary Fig. S2B). We determined the IC_50_ concentrations of the original compounds and of these five analogues. The parent compounds Z637352244 and Z637352642 exhibited IC_50_ values of 22 and 223 μM, respectively (Supplementary Fig. S2C). Three analogues, Z56785964, Z637450230 and Z56786187, had IC_50_ values between 13–24 μM, whereas two analogues, Z2239054061 and Z637352638, had IC_50_ values of 6.7 and 7.5 μM, respectively (Supplementary Fig. S2C), representing a significant improvement over the parent compounds.

To further characterize the diamino-quinazoline and nitrile compounds, we examined their effect on the melting temperature of M^pro^ using a thermal shift assay (TSA). Briefly, M^pro^, at a final concentration of 1 μM, was incubated for 20 min with the inhibitors, at a final concentration of 20 μM, and the melting temperature of M^pro^ was determined. Compounds that bind to M^pro^ should enhance its melting temperature^31^. Indeed, GC376 increased the melting temperature of M^pro^ by 19 °C (Supplementary Fig. S3A). Surprisingly, the parent nitrile-containing compounds decreased the melting temperature of M^pro^, as did all their analogues, except for analogue Z637450230, which did not affect the melting temperature of M^pro^ (Supplementary Fig. S3A). The diamino-quinazoline compound Z1037455358 also decreased the melting temperature of M^pro^ (Supplementary Fig. S3B).

The decrease in the melting temperature of M^pro^ by the above compounds was of concern, as it could indicate that these compounds were destabilizing M^pro^. Compounds that denature proteins non-specifically should lose inhibitory activity in the presence of excess carrier protein, since the latter can serve as a sink for compound sequestration. Indeed, the parent nitrile-containing compound Z637352244, its analogues Z56786187 and Z637450230, and the diamino-quinazoline compound Z1037455358, all lost activity, when the protease assay was performed in the presence of 1 μg cell lysate (Supplementary Fig. S2D). In contrast, GC376 maintained inhibitory activity in the presence of lysate and, interestingly, so did the dihydro-quinolinones compounds ZINC000636416501 and ZINC000373659060 (Supplementary Fig. S2D).

### Characterization of the dihydro-quinolinone compounds

Compounds ZINC000636416501 and ZINC000373659060 are related to each other and contain a dihydro-quinolinone core (Fig. 1B). Encouraged by the fact that the activity of these two compounds was not affected by the presence of cell lysate, we obtained 157 analogues (Supplementary Table 7) and examined their ability to inhibit M^pro^. Three analogues were significantly more potent than the parent compounds (Supplementary Fig. S4A). Specifically, compounds Z228770960, Z393665558 and Z225602086 had IC_50_ values of 4, 6 and 7.4 μM, respectively, whereas the parent compounds ZINC000373659060 and ZINC000636416501 had IC50 values of 58 and 93 μM, respectively (Supplementary Fig. S4B). Importantly, all three analogues retained their inhibitory activity against M^pro^ in the presence of cell lysate (Supplementary Fig. S4C).

The parent ZINC000636416501 and ZINC000373659060 compounds and their three active analogues were then examined for their ability to modulate the melting temperature of M^pro^ using the thermal shift assay. The parent compounds did not affect the melting temperature of M^pro^ (Supplementary Fig. S4D). However, the analogues increased the melting temperature of M^pro^ with the most active analogue, Z228770960, inducing an increase of 1.2 °C (Supplementary Fig. S4E). These results are consistent with the analogues forming stable complexes with M^pro^.

To identify even more potent compounds, we performed a second round of analogue synthesis, using the first-round analogues Z228770960, Z393665558 and Z225602086 as starting points for structure similarity searches. A total of 113 second-round analogues were selected for on-demand synthesis (Supplementary Table 8), of which four were as potent or more potent than the analogues from the first round (Fig. 1C and Supplementary Fig. S5A). Specifically, compounds Z222979552, Z228166018, Z222977344 and Z222978028 had IC_50_ values of 1.0, 1.6, 2.0 and 5.8 μM, respectively, which inhibitory activity they retained in the presence of cell lysate (Fig. 1D and Supplementary Fig. S5B). The above four analogues also increased the melting temperature of M^pro^ by up to 1.2 °C (Fig. 1E and Supplementary Fig. S5C).

The first and second rounds of analogue synthesis resulted in about 15-fold and four-fold improvements in the IC_50_ values, respectively. However, additional chemical diversity of structures that we could gain from further rounds of compound similarity searches was limited. We therefore decided to characterize the most potent dihydro-quinolinone M^pro^ inhibitor, Z222979552 (IC_50_ = 1.0 μM), by determining its crystal structure in complex with M^pro^ and its antiviral activity in cell-based assays.

### Structure of a dihydro-quinolinone inhibitor in complex with M^pro^

**Figure 2:**
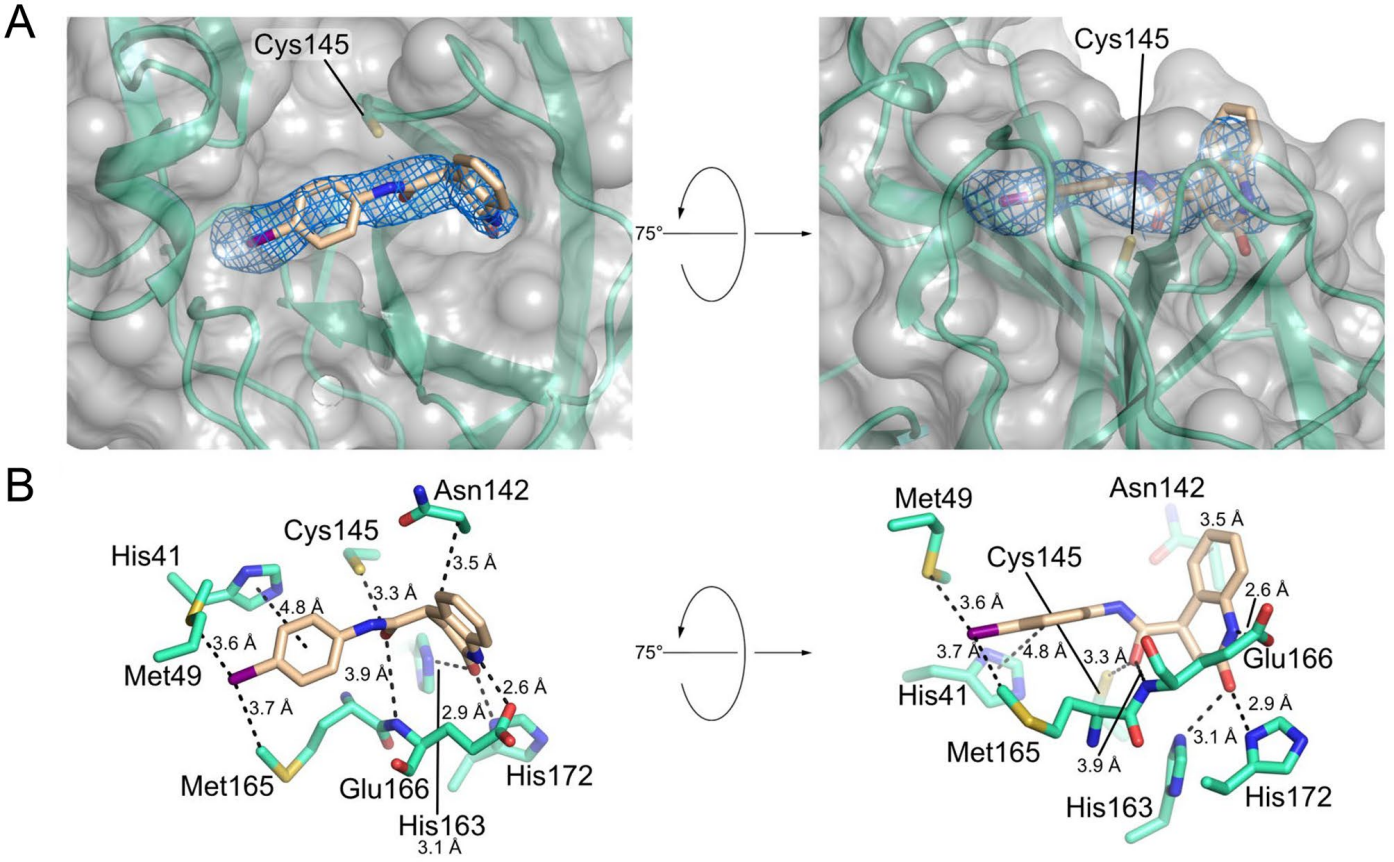
Crystal structure of M^pro^ in complex with the dihydro-quinolinone compound Z222979552. (**A**) Surface representation of SARS-CoV-2 M^pro^ with secondary structure elements colored green and compound Z222979552 shown as a stick model. The omit electron density map corresponding to the ligand compound is shown (σ = 1.0). (**B**) Stick model of compound Z222979552 and of select residues at the active site of M^pro^ showing the key interactions between the protease and its inhibitor.

The crystal structure of M^pro^ in complex with compound Z222979552 was solved at a resolution of 2.5 Å and shows the compound in the active site of M^pro^ (Fig. 2A; Table 1). Unlike the previously described high-affinity M^pro^ inhibitors, compound Z222979552 did not form a covalent bond with M^pro^. Rather, binding was mediated by hydrogen bonds, pi-stacking and hydrophobic interactions. Hydrogen bonds were observed between the dihydro-quinolinone group and the side chains of Glu166, His163 and His172, as well as between the carbonyl group of the compound and the thiol group of Cys145 and the main chain of Glu166 (Fig. 2B). A T-type pi-stacking interaction was observed between the benzene ring and His41, while the dihydro-quinolinone group, the benzene group and the iodine atom participated in hydrophobic interactions with Asn142, Met49 and Met165, respectively (Fig. 2B).

**Table 1:** X-ray diffraction data and phasing and refinement statistics.

	M^pro^ in complex with Z222979552^a^
**Data collection**	
# Crystals/# datasets	1/1
Space group	C 1 2 1
Unit cell dimensions	
*a*, *b*, *c* (Å)	114.61, 53.79, 45.44
*α*, *β*, *γ* (°)	90.00, 100.98, 90.00
# of reflections	31,270 (2882)
# of unique reflections	9258 (912)
R_merge_ (%)	0.17 (2.37)
cc_1/2_	0.98 (0.21)
I/σI	8.38 (0.64)
Completeness	98.65 (97.33)
Redundancy	3.40 (3.20)
**Refinement**	
Resolution (Å)	48.53–2.50
No. of reflections	18,044 (1759)
R_work_/R_free_	0.2255/0.2783
Bond lengths (Å)	0.002
Bond angles (°)	0.45
No. of non-hydrogen atoms	2383
Protein	2354
Ligand	21
Solvent	8
***B*-factors**	
Protein	87.30
Ligand	138.11
Solvent	74.72

^a^Values in parentheses are for the highest resolution shell.

### Inhibition of viral replication by a dihydro-quinolinone M^pro^ inhibitor

**Figure 3:**
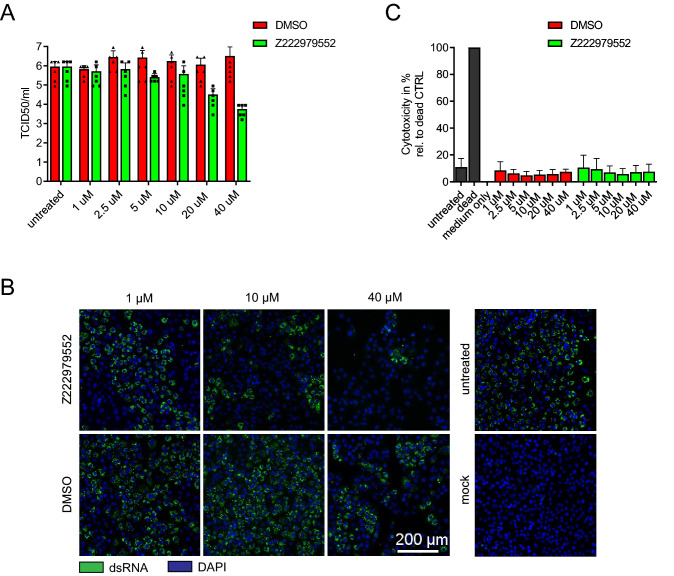
Compound Z222979552 reduces SARS-CoV-2 replication in Vero E6 cells in a dose-dependent manner. (**A**) SARS-CoV-2 titers upon treatment with compound Z222979552 or DMSO control. Viral titers are displayed as fifty-percent tissue culture infective dose (TCID_50_)/ml 24 h post-infection. TCID_50_ values correspond to the viral titers (log_10_ scale) required to kill fifty-percent of infected host cells. The experiment was performed in triplicate and the results are shown as means and standard deviations. (**B**) Immunofluorescence staining of infected Vero E6 cells for double-stranded RNA (dsRNA). The cells were treated with compound Z222979552 or DMSO and were stained 24 h post-infection. Green, dsRNA; blue, DAPI. One representative image out of three biological replicates is shown. (**C**) Z222979552-mediated cytotoxicity, determined using Vero E6 cells treated with the compound or DMSO for 24 h. The experiment was performed in triplicate and the results are shown as means and standard deviations.

To further characterize compound Z222979552, we examined whether it could inhibit SARS-CoV-2 replication in Vero E6 cells. Consistent with its ability to inhibit M^pro^ in vitro, Z222979552 suppressed SARS-CoV-2 replication in Vero E6 cells, resulting in a more than a 100-fold decrease of SARS-CoV-2 titers at the highest concentration tested (Fig. 3A). In a second assay we monitored by immunofluorescence the presence of viral double-stranded RNA (dsRNA) in Vero E6 cells infected with SARS-CoV-2. Treatment of the cells with Z222979552 prevented the formation of dsRNA intermediates of SARS-CoV-2 RNA synthesis (Fig. 3B). We also performed a cytotoxicity assay with non-infected Vero E6 cells and observed that Z222979552 is not cytotoxic (Fig. 3C). Finally, we validated the cell-based assays using remdesivir as a positive control (Supplementary Fig. S6). Taken together, the above findings indicate that compound Z222979552 has antiviral activity in cells.

## Discussion

COVID-19 has had a significant impact on our society. The vaccines have significantly curtailed the spread of the pandemic^32^, but challenges still remain regarding vaccine acceptance by the public and durability of vaccine efficacy over time^33,34^. Thus, there continues to be an urgent need for novel medicines to treat COVID-19 and M^pro^ is considered a valuable target for the development of SARS-CoV-2 antivirals, because it is required for viral replication^5–7^. Equally importantly, M^pro^ is highly conserved in evolution^5^, which means that inhibitors developed against M^pro^ of one coronavirus may inhibit replication of multiple members of the coronavirus family. Indeed, SARS-CoV-2 M^pro^ inhibitors entering the clinic were derived from compounds that were originally developed as inhibitors of SARS-CoV-1 M^pro^, but whose development was, unfortunately, halted, when the SARS-CoV-1 epidemic waned^14,15,23^.

All the inhibitors described here were obtained from the REAL Space or the ZINC chemical compound libraries, which together encompass more than 20 billion make-on-demand compounds^29,30^. Despite their large size, these two libraries represent a very small part of the universe of chemical space. Therefore, we anticipate that the compounds that we have identified can serve as starting points for medicinal chemistry efforts, particularly because these compounds have drug-like features and activity in cell-based assays. Moreover, their mechanism of action is understood from the crystal structure of compound Z222979552 in complex with M^pro^.

One interesting feature of the inhibitors described here is that they are non-covalent, in contrast to the vast majority of previously described M^pro^ inhibitors, including the PF-07321332 inhibitor, which form covalent bonds with the catalytic cysteine. The therapeutic significance of having a non-covalent M^pro^ inhibitor is unclear at this moment, but the possibility of developing such inhibitors to complement the covalent inhibitors can only offer more therapeutic opportunities.

## Methods

### Protein expression and purification of SARS-CoV-2 M^pro^

The M^pro^ construct^8^ provided by Rolf Hilgenfeld was transformed into *E. coli* strain BL21-Gold (DE3) (Agilent). Transformed clones were picked to prepare pre-starter cultures in 2 mL YT medium with ampicillin (100 μg/ml), at 37 °C for 8 h. The pre-starter culture was then inoculated into fresh 120 mL YT medium with ampicillin (100 μg/ml) and incubated at 37 °C overnight. The next day, the starter culture was inoculated into 1,600 mL YT medium with ampicillin (100 μg/ml) and incubated at 37 °C until OD_600_ reached a value between 0.6 and 0.8. 1 mM isopropyl-D-thiogalactoside (IPTG) was then added to induce the overexpression of M^pro^ at 30 °C for 5 h. The bacteria were harvested by centrifugation at 8260 × g, 4 °C for 15 min, resuspended in Binding Buffer (25 mM BTP [pH 6.8]; 300 mM NaCl; 2 mM DTT; 1 mM EDTA; 3% DMSO) and then lysed using an Emulsiflex-C3 homogenizer (Avestin). The lysate was clarified by ultracentrifugation at 137,088 × g, 4 °C for 1 h and loaded onto a HisTrap FF column (Cytiva) using an Äkta protein purification system (Cytiva). When all the supernatant containing M^pro^ had passed through the column, the column was washed with 80 mL binding buffer to remove non-specifically bound proteins and then M^pro^ was eluted using an imidazole gradient (0–500 mM) in Binding Buffer. The M^pro^ fractions were concentrated using 3 kDa Amicon Ultra Centrifugal Filters (Merck Millipore) and the M^pro^ protein was further purified by size exclusion chromatography using a HiLoad Superdex 200 column (Cytiva) attached to a SMART protein purification system (Pharmacia).

### Compounds

We initially selected for on-demand synthesis 485 compounds that were identified by two in silico screening studies as putative M^pro^ inhibitors; these compounds are referred to as parent compounds (Supplementary Tables 1–4). After evaluating the activity of the parent compounds in vitro, we selected an additional 686 compounds that were analogues of the few active parent compounds (Supplementary Tables 5–8). All the above compounds were purchased from Enamine, their purity was ≥ 90% and they were synthesized on-demand. The compounds were dissolved in DMSO at a concentration of 2 mM and were stored at -20 °C. GC376 was purchased from BPSBioscience.

### SARS-CoV-2 M^pro^ protease activity assay

M^pro^ protease assays were performed in duplicate in Falcon 384-well optilux flat bottom, TC-treated microplates (Corning) in a final volume of 10 μL. M^pro^, at a final concentration of 100 nM, was preincubated for 20 min at room temperature (RT) with the compounds in assay buffer (5 mM HEPES pH 7.5, 0.1 mg/mL BSA, 0.01% Triton, 2 mM DTT) under gentle agitation. The FRET substrate, HiLyte-Fluor488-ESATLQSGLRKAK(QXL520)-NH2 (Eurogentec), was then added at a final concentration of 500 nM and incubated for 2 min at RT with gentle agitation prior to the start of fluorescence measurement. Compounds and FRET substrate were dispensed with an acoustic liquid dispenser (Gen5-Acoustic Transfer System; EDC Biosystems). The fluorescence intensity was measured kinetically for 7 cycles, every 10 min at 22 °C, using a Spark 10 M microplate reader (Tecan) and excitation and emission wavelengths of 485 and 528 nm, respectively.

### SARS-CoV-2 M^pro^ thermal shift binding assay

Thermal shift assays were performed in duplicate in LightCycler 480 multiwell plates 96, white (Roche) in a final volume of 20 μL. M^pro^ protease, at a final concentration of 1 μM, was preincubated for 20 min at room temperature (RT) under gentle agitation with the compounds (final concentration: 20 μM) in assay buffer (10 mM HEPES pH 7.5; 150 mM NaCl). Protein unfolding was monitored with 5X SYPRO Orange (Sigma) binding dye. Compounds and SYPRO Orange were dispensed with an acoustic liquid dispenser (Gen5-Acoustic Transfer System; EDC Biosystems). Fluorescence (excitation wavelength: 465 nm; emission wavelength: 580 nm) was measured over a temperature gradient ranging from 20 to 95 °C, with incremental steps of 0.05 °C/s and 11 acquisitions per °C. The melting curves and peaks were obtained using the melting temperature (Tm) calling analysis of the LightCycler 480 Software (release 1.5.1.62; Roche; https://lifescience.roche.com/en_ch/brands/realtime-pcr-overview.html#software).

### Crystallization and model building

Purified M^pro^ was concentrated to 5.6 mg/mL and crystallized using the hanging vapor diffusion method. Plate-shaped crystals grew in star-like clusters within one week in drops mixed in a 1:1 ratio with reservoir solution (0.1 M Tris [pH 8.0], 25% (v/v) PEG-3350 and 0.2 M LiCl) at 293 K. For soaking and cryoprotection, the crystal clusters were moved into 2 μL reservoir solution supplemented with 25% ethylene glycol. Then, 0.4 μL inhibitor solution was added resulting in a final compound concentration of 10 mM. Soaking was allowed to proceed for 2 h, after which single crystals were broken off the clusters and flash-frozen in liquid nitrogen. Diffraction data was collected at the Swiss Light Source (SLS), beamline PXI (X06SA) with λ = 1.000 Å at T = 100 K. The data were processed using the XDS package^35^ up to a resolution of 2.5 Å. The structure was solved using Phaser^35^ from the Phenix suite (v.19.1.) with 6wqf as a search model. In Phenix, the model was iteratively refined using Phenix Refine^36^ with manual adjustments done in Coot6 (v.0.9.3). The three-dimensional model and molecular restraints of the ligand were generated using eLBOW7 from the Phenix suite^37^. According to the Ramachandran statistics for the final model 95.38% of residues are in favored regions, 3.96% are in allowed regions, and 0.77% of residues are outliers. All figures concerning structural data were prepared with OpenSource Pymol by Schrodinger (v.1.20; https://github.com/schrodinger/pymol-open-source).

### Viral replication assays

Vero E6 cells (kindly provided by Doreen Muth, Marcel Müller and Christian Drosten, Charité, Berlin, Germany) were propagated in Dulbecco's modified EMEM (DMEM), supplemented with 10% heat-inactivated fetal bovine serum, 1% nonessential amino acids, 100 µg/mL streptomycin, 100 IU/mL penicillin and 15 mM HEPES at 37 °C in a humidified incubator with 5% CO2. SARS-CoV-2 (SARS-CoV-2/München-1.1/2020/929, kindly provided by Daniela Niemeyer, Marcel Müller and Christian Drosten) passage 1 was used for infection of the cells.

Two million Vero E6 cells were plated per well of a 96-well plate; 24 h later, the cells were infected with SARS-CoV-2/München-1.1/2020/929 passage 1 at an MOI of 0.1 for 1 h at 37 °C and then washed 3 times with PBS. Z222979552 and remdesivir (or respective volumes of DMSO) were added to cells in following concentrations: 0, 1, 2.5, 5, 10, 20 and 40 µM. 24 h post-infection infectious supernatant was serially diluted and the 50% tissue culture infectious dose (TCID_50_) per ml was determined 96 h later using the Spearman-Kärber algorithm, as previously described^38^. Cytotoxic effects of Z222979552, remdesivir or the corresponding DMSO volumes were determined using the CytoTox 96 Non-Radioactive Cytotoxicity Assay (Promega).

Vero E6 cells infected with SARS-CoV-2 were fixed with 4% formalin and then permeabilized in PBS supplemented with 50 mM NH_4_Cl, 0.1% (w/v) Saponin and 2% (w/v) Bovine Serum Albumin. The cells were stained using a mouse monoclonal antibody against dsRNA (SCICONS, clone J2) and Alexa-Fluor 488-labeled donkey-anti mouse IgG (H + L) (JacksonImmuno) secondary antibody. Images were acquired with an EVOS FL Auto 2 Imaging System, using a 10 × objective lens and processed using Fiji software packages^39^ version 1.53j (https://imagej.net/software/fiji/downloads) and assembled with the FigureJ plugin^40^ version 1.36 (https://imagejdocu.list.lu/plugin/utilities/figurej/start).

## Supplementary Information

Supplementary Tables.

Supplementary Figures.

## Data Availability

Atomic coordinates and structure factors for the crystal structure of M^pro^ in complex with Z222979552 have been deposited in the Protein Data Bank under accession code 7P2G.
