# Supplementary material for: Non-covalent SARS-CoV-2 Mpro inhibitors developed from in silico screen hits
Source: Sci Rep. 2022 Feb 15;12:2505. doi: 10.1038/s41598-022-06306-4 (PMC8847420; doi:10.1038/s41598-022-06306-4)
Supplement: Supplementary file 2 — Supplementary Figures. [file 41598_2022_6306_MOESM2_ESM.pdf]

Supplementary Figures

Non-covalent SARS-CoV-2 M^{pro} inhibitors developed from in silico screen hits

Rossetti, G. G., *et al.*

Supplementary Figure S1

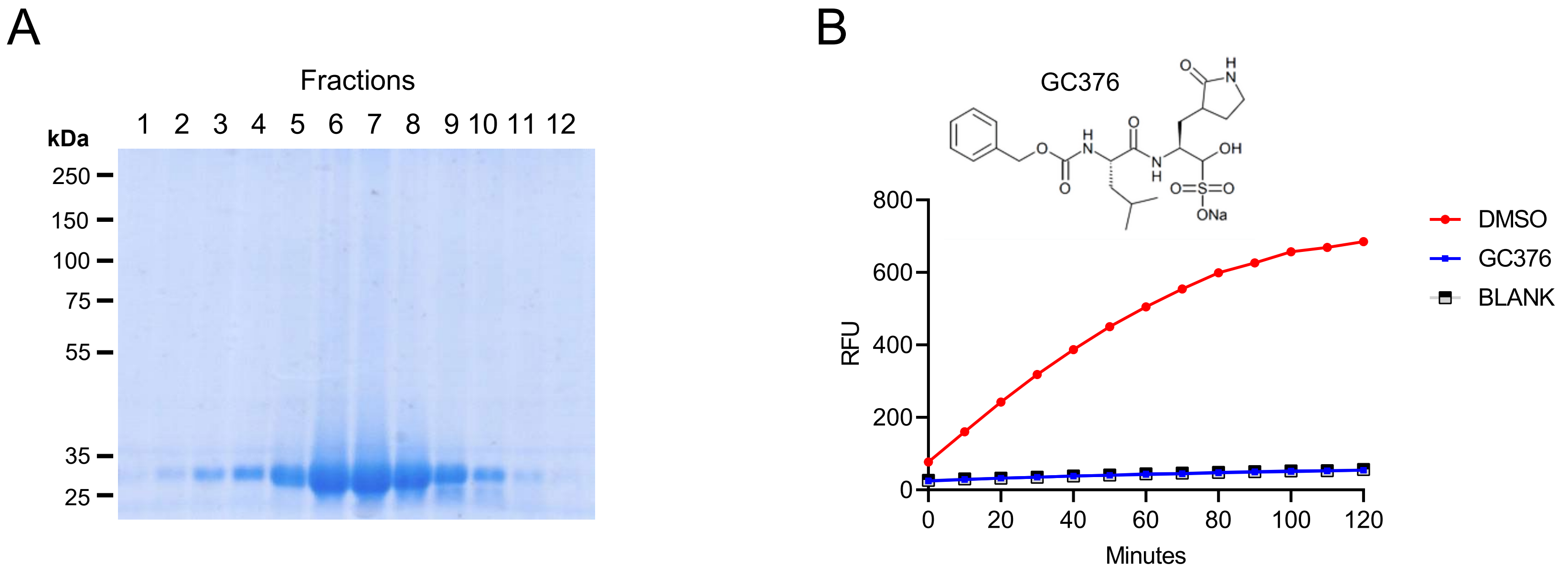

Supplementary Figure S1. Validation of *in silico* screening hits by an *in vitro* protease assay.

(A) Purification of His-tagged M^{pro} by affinity chromatography. Protein fractions eluted from a nickel-charged Sepharose (HisTrap FF) column by an imidazole gradient were examined by SDS-PAGE. The positions of migration of the molecular weight markers are shown on the left. M^{pro} migrates at about 30 kDa, consistent with its molecular weight.

(B) The protease activity of M^{pro} was monitored by a FRET assay, as described in the text. In this experiment, fluorescence was measured every 10 min over a period of 120 min. GC376, a previously described M^{pro} inhibitor, whose structure is shown, was examined at a final concentration of 40 μM. Blank, reaction omitting M^{pro}; RFU, relative fluorescence units.

Supplementary Figure S2

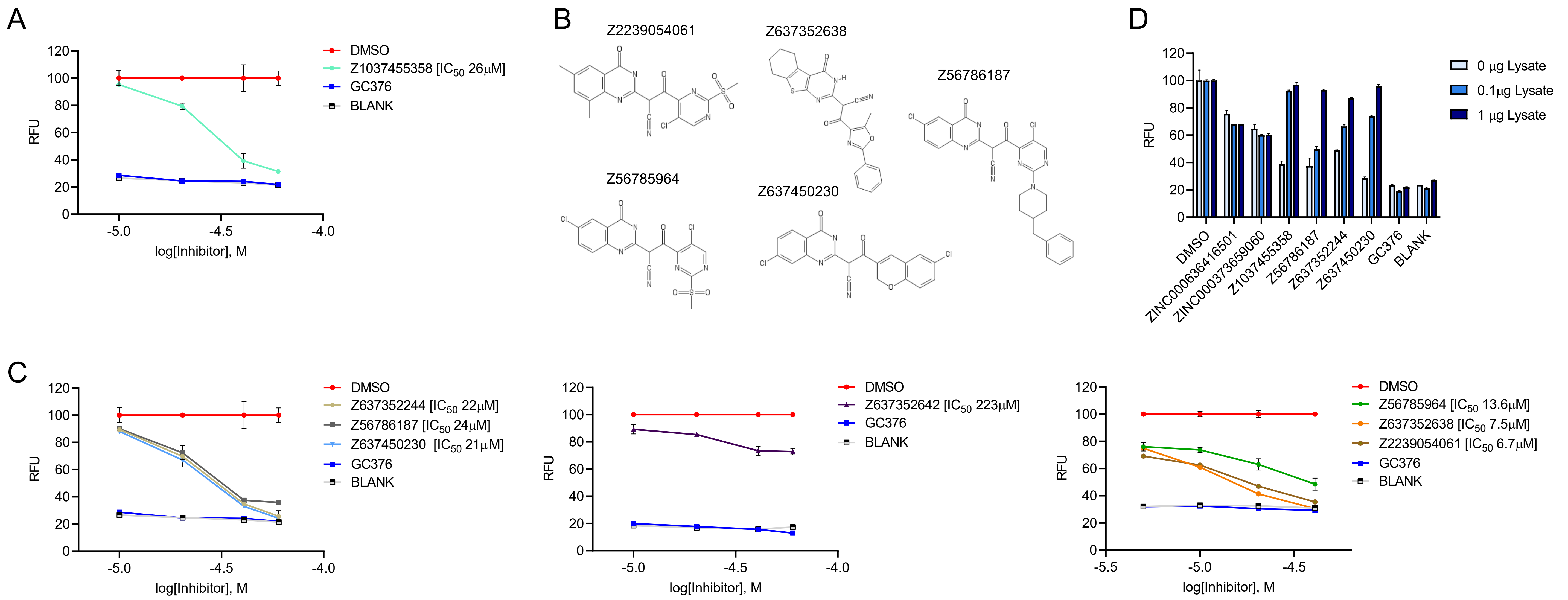

Supplementary Figure S2. M^{pro} protease inhibitory activity of compounds containing diamino-quinazoline or nitrile groups.

(A) Dose-response curve for the diamino-quinazoline compound Z1037455358 examined at 10, 20, 40 and 60 µM final compound concentrations. The protease assay was performed, as described in Supplementary Fig. S1B, except that fluorescence was measured every 10 min over a period of 60 min. RFU, relative fluorescence units.

(B) Chemical structures of the most active analogues containing a nitrile group.

(C) Dose-response curves for compounds Z637352244, Z637352642 and their analogues examined at 10, 20, 40 and 60 µM or 5, 10, 20 and 40 µM final compound concentrations, as indicated.

(D) Effect of cell lysate on the M^{pro} inhibitory activity of selected compounds. The M^{pro} FRET protease assay was performed in the absence or presence of 0.1 or 1 µg cell lysate. The final concentration of the compounds was 40 µM.

Supplementary Figure S3

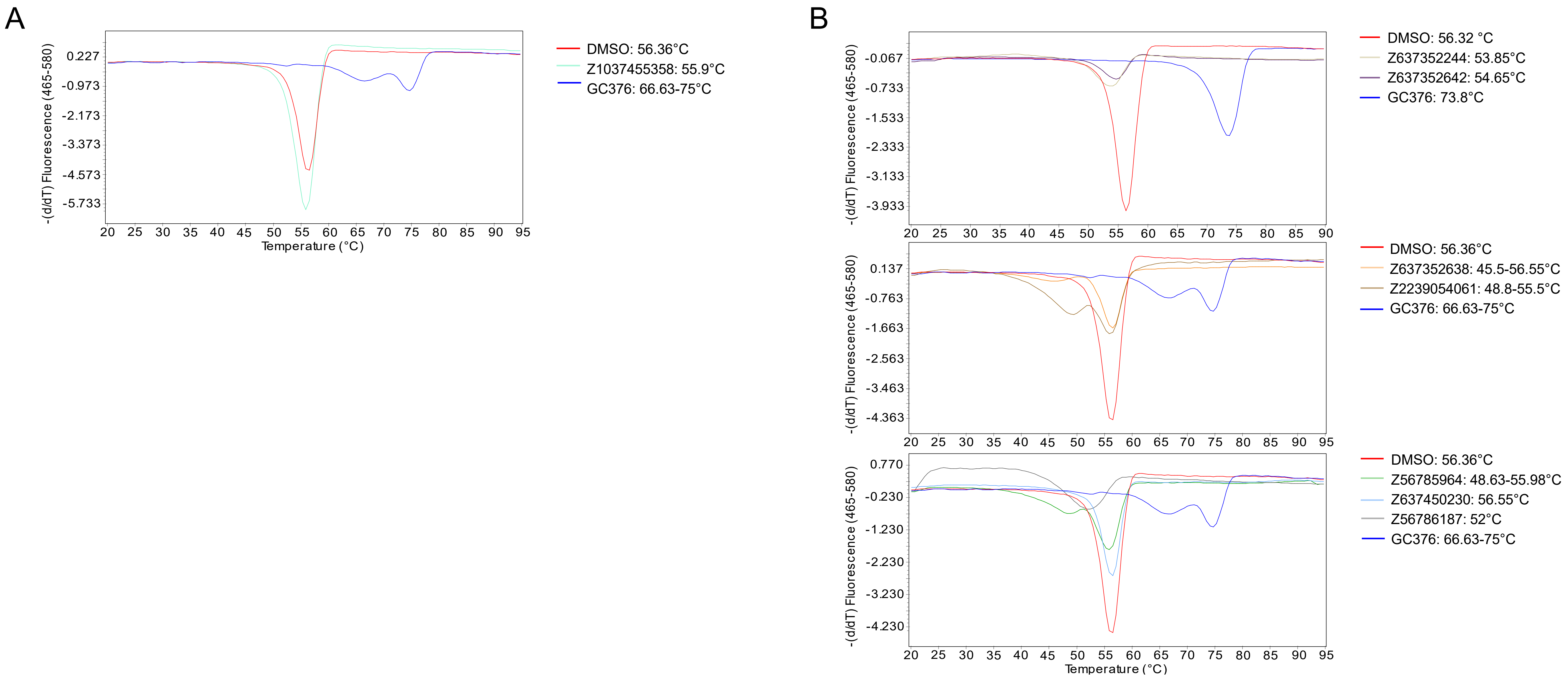

Supplementary Figure S3. Effect of compounds containing diamino-quinazoline or nitrile groups on the melting temperature of M^{pro}. Thermal shift assays were performed in the presence of DMSO or 20 μM M^{pro} inhibitors. The graphs show the melting curve derivatives, which were used to calculate the melting temperature of M^{pro}.

(A) Thermal shift assay for the diamino-quinazoline compound Z1037455358.

(B) Thermal shift assay for the compounds containing nitrile groups.

Supplementary Figure S4

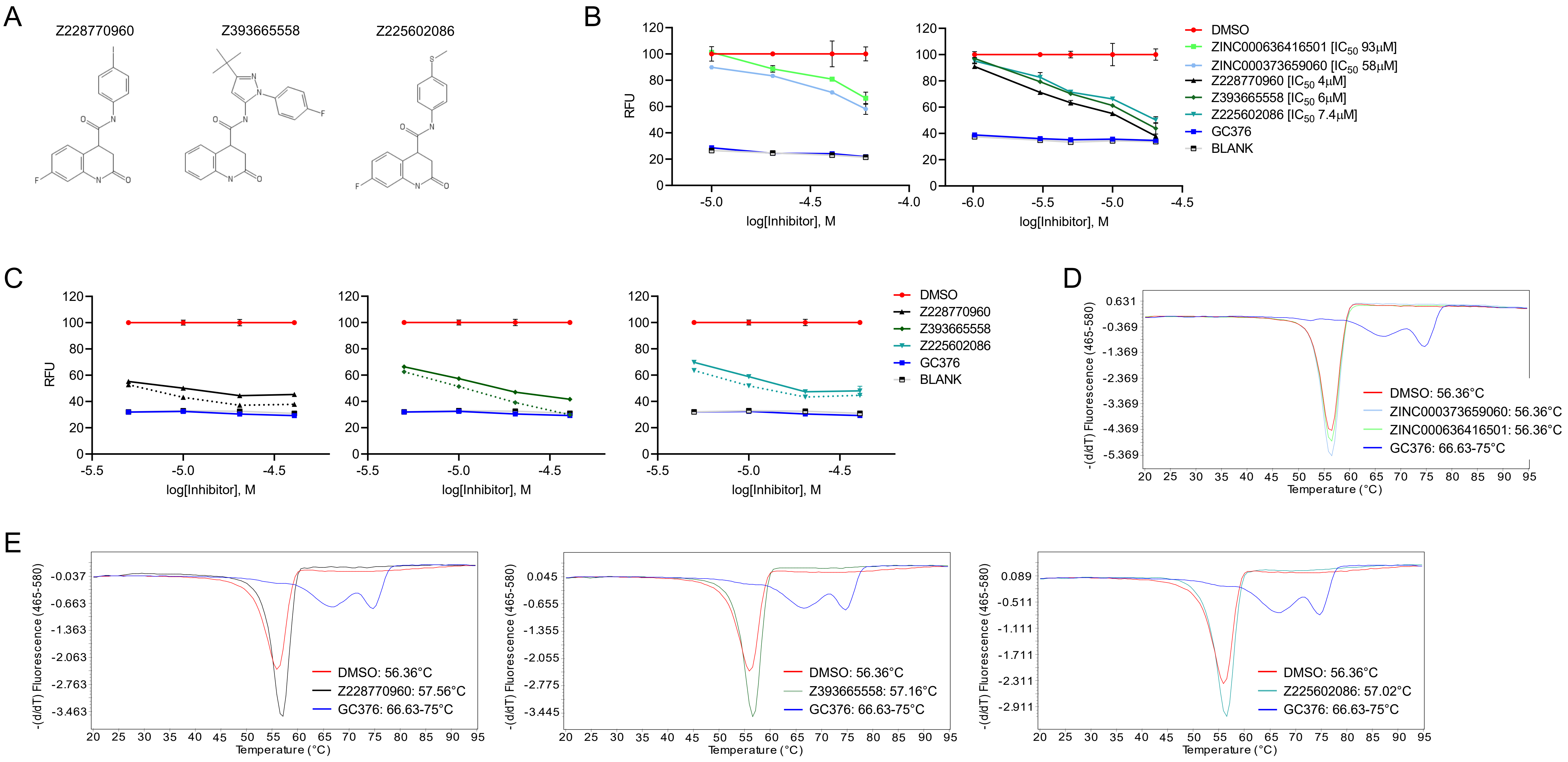

Supplementary Figure S4. Characterization of the original dihydro-quinolinone compounds and first-round analogues.
(A) Chemical structure of the most active first-round dihydro-quinolinone analogues.
(B) Dose-response curves of the parent compounds ZINC000636416501 and ZINC000373659060 (10, 20, 40, 60 µM) and their most active first-round analogues (1, 3, 5, 10, 20, 40 µM) in the M^{pro} protease assay performed, as described in Supplementary Fig. S2A. RFU, relative fluorescence units.
(C) Dose-response curves of the most active first-round dihydro-quinolinone analogues (5, 10, 20, 40 µM) in absence (solid lines) or presence (dashed lines) of 0.1 µg protein lysate.
(D) Thermal shift assays performed in the presence of DMSO or 20 µM of the parent dihydro-quinolinone compounds. The graphs show the derivatives of the melting curves that were used to calculate the melting temperature of M^{pro}.
(E) Thermal shift assays performed in the presence of DMSO or 20 µM of the most active first-round dihydro-quinolinone analogues.

Supplementary Figure S5

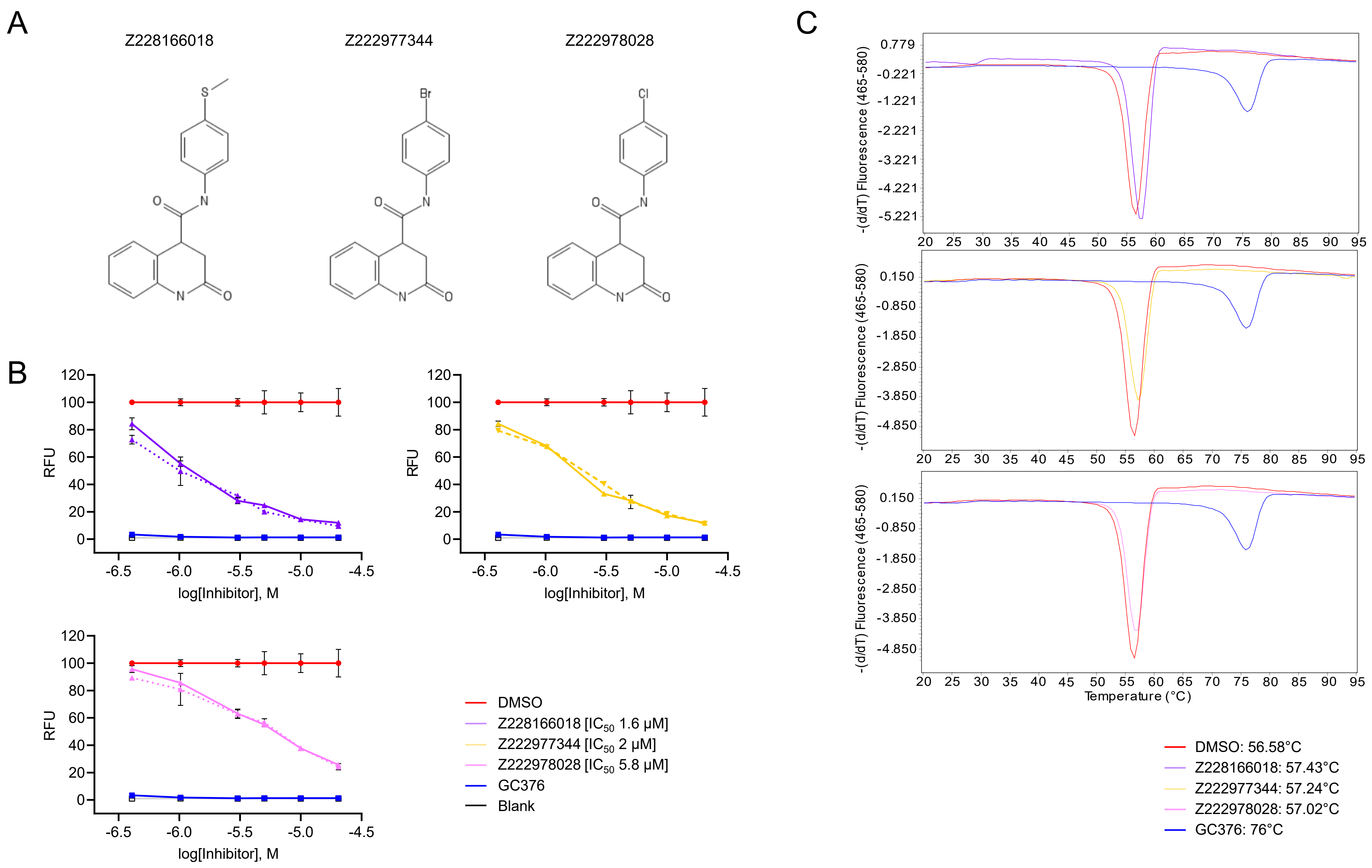

Supplementary Figure S5. Characterization of selected second-round dihydro-quinolinone analogues.

(A) Chemical structure of selected second-round dihydro-quinolinone analogues (see also Fig. 1).

(B) Dose-response curves for second-round analogues Z228166018, Z222977344 and Z222978028 examined at 0.4, 1, 3, 5, 10 and 20 μM final compound concentrations, in absence (solid lines) or presence (dashed lines) of 0.1 μg protein lysate. The M^{pro} protease assay was performed, as described in Supplementary Fig. S2A. RFU, relative fluorescence units.

(C) Thermal shift assays performed in the presence of DMSO or 20 μM of selected second-round dihydro-quinolinone analogues. The graphs show the derivatives of the melting curves that were used to calculate the melting temperature of M^{pro}.

Supplementary Figure S6

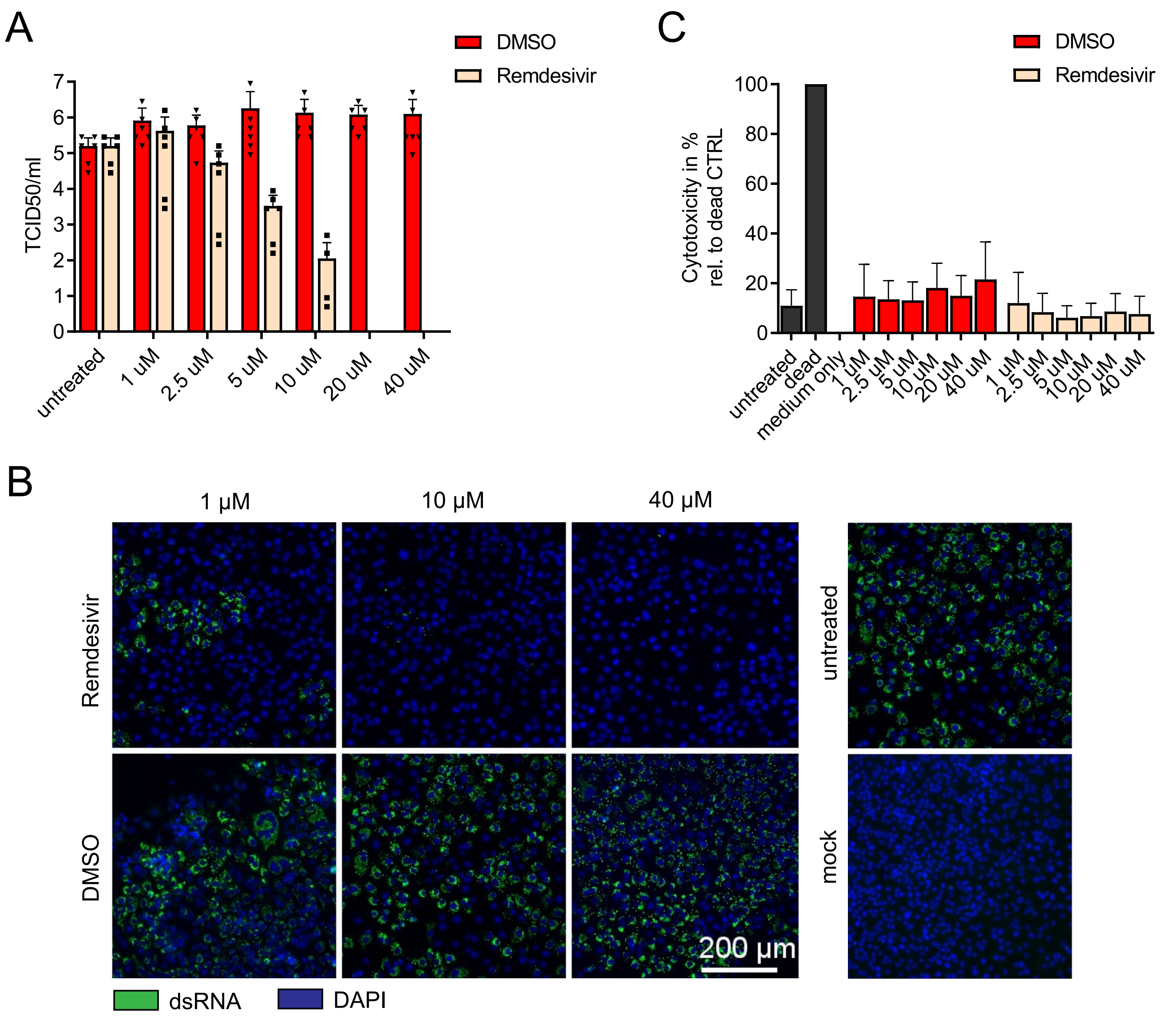

Supplementary Figure S6. Validation of viral replication assays in Vero E6 cells using remdesivir as a positive control.

(A) SARS-CoV-2 titers upon treatment with remdesivir or DMSO control. Viral titers are displayed as fifty-percent tissue culture infective dose ($TCID_{50}$)/ml 24 h post-infection. $TCID_{50}$ values correspond to the viral titers (log_{10} scale) required to kill fifty percent of infected host cells. The experiment was performed in triplicate and the results are shown as means and standard deviations.

(B) Immunofluorescence staining of infected Vero E6 cells for double-stranded RNA (dsRNA). The cells were treated with remdesivir or DMSO and were stained 24 h post-infection. Green, dsRNA; blue, DAPI.

(C) Remdesivir-mediated cytotoxicity, determined using Vero E6 cells treated with the compound or DMSO for 24 h. The experiment was performed in triplicate and the results are shown as means and standard deviations.
